# Development of bioluminescent *Salmonella *strains for use in food safety

**DOI:** 10.1186/1471-2180-8-10

**Published:** 2008-01-22

**Authors:** Attila Karsi, Kevin Howe, Tasha B Kirkpatrick, Robert Wills, R Hartford Bailey, Mark L Lawrence

**Affiliations:** 1 Department of Basic Sciences, College of Veterinary Medicine, Mississippi State University, P.O. Box 6100, Mississippi State, MS 39762-6100, USA; 2 Department of Pathobiology and Population Medicine, College of Veterinary Medicine, Mississippi State University, P.O. Box 6100, Mississippi State, MS 39762-6100, USA

## Abstract

**Background:**

*Salmonella *can reside in healthy animals without the manifestation of any adverse effects on the carrier. If raw products of animal origin are not handled properly during processing or cooked to a proper temperature during preparation, salmonellosis can occur. In this research, we developed bioluminescent *Salmonella *strains that can be used for real-time monitoring of the pathogen's growth on food products. To accomplish this, twelve *Salmonella *strains from the broiler production continuum were transformed with the broad host range plasmid pAK*lux*1, and a chicken skin attachment model was developed.

**Results:**

*Salmonella *strains carrying pAK*lux*1 constitutively expressed the *luxCDABE *operon and were therefore detectable using bioluminescence. Strains were characterized in terms of bioluminescence properties and plasmid stability. To assess the usefulness of bioluminescent *Salmonella *strains in food safety studies, we developed an attachment model using chicken skin. The effect of washing on attachment of *Salmonella *strains to chicken skin was tested using bioluminescent strains, which revealed the attachment properties of each strain.

**Conclusion:**

This study demonstrated that bioluminescence is a sensitive and effective tool to detect *Salmonella *on food products in real-time. Bioluminescence imaging is a promising technology that can be utilized to evaluate new food safety measures for reducing *Salmonella *contamination on food products.

## Background

*Salmonella enterica*, with over 2000 different serovars, is indigenous to the gastrointestinal tracts of many mammals, birds, and reptiles, usually in low levels. *Salmonella *can be pathogenic in these animals if it reaches certain numbers in vivo. However, moribund animals are usually culled before they reach slaughter. Therefore, the food safety problem relative to humans is that *Salmonella *can be carried into the processing plant in healthy asymptomatic animals. If cross-contamination occurs during the slaughter process, *Salmonella *can then be transferred to carcasses that were previously uncontaminated. A significant increase in the number of *Salmonella *positive broilers between exiting the scalding water immersion tank and exiting the crop extractor demonstrates how in-plant cross-contamination can occur [1,2]. When carcasses were sampled at 6 points in processing, an increase occurred in the presence of *Salmonella *from 19% pre-scald to 36.9 % post-chill (after exiting the immersion chill tank).

Regardless of whether the source of contamination was pre-harvest or during processing, *Salmonella *is difficult to remove from carcasses due to its ability to adhere to chicken skin and endure the stages of processing [3]. Laboratory research, as well as in-plant trials, has demonstrated this relationship [4-7]. Therefore, persistence of *Salmonella *within the processing plant may be partially explained by interactions between chicken skin and *Salmonella*. Chemical treatments have been developed and shown to be effective under controlled conditions in the reduction of *Salmonella *levels on broiler carcasses or skin [8-11]. In spite of these efforts, *Salmonella *is yet to be eliminated from the process.

When testing the efficacy of different antimicrobial compounds, conventional cultural techniques are typically used. The product is sampled, microbiological culturing protocols are followed, and 48 to 72 hours later the results are known. Bioluminescence imaging (BLI) is a technique that can be used for real-time quantification and tracking of live bacteria in hosts [12-15]. To enable BLI, bacteria are tagged with bacterial luciferase, which catalyzes the oxidation of a long-chain aldehyde and FMNH_2 _to cause emission of visible light [16-19]. *Salmonella *strains constitutively expressing bacterial luciferase have no significant alterations in phenotype, including growth kinetics [20,21] or biochemical, serological, or structural phenotypes [20,22]. Advantages of the bacterial luciferase reporter system include negligible background [23], no toxic or phenotypic effects from accumulation of signal [24], real-time detection, and no need for addition of an exogenous substrate. In addition, bioluminescence from bacterial luciferase correlates well with the amount of luciferase protein and *lux *mRNA [25,26], bacterial plate counts [20,27], and intracellular numbers of bacteria in cell culture [28].

In this research, our aim was to develop twelve bioluminescent *Salmonella enterica *strains that can be used for real-time monitoring of the pathogen's growth on food products. Our study is unique in that it includes multiple *Salmonella *field strains isolated from the broiler production continuum, including post hatchery, prior to harvest, arrival at the plant, pre-chill tank, and post-chill tank.

## Results and discussion

### Bioluminescent *Salmonella* strains

pAK*lux*1 was transferred to twelve *Salmonella *strains that we isolated in a study on the poultry production and processing continuum. The strains represented the twelve most commonly isolated serovars from our study. This result compared favorably with a previous report where transformation of a *lux *plasmid into *Salmonella *isolates from a poultry processing plant was only successful for one isolate out of seven attempted [20]. Expression of the *luxCDABE *operon, which encodes bacterial luciferase, was driven by the *lacZ *promoter on pAK*lux*1. Because *Salmonella *does not have *lacI*^q ^in its chromosome, it constitutively expresses the *lacZ *promoter on pAK*lux*1 and hence produces continuous light while it is alive and metabolically active.

**Figure 1:**
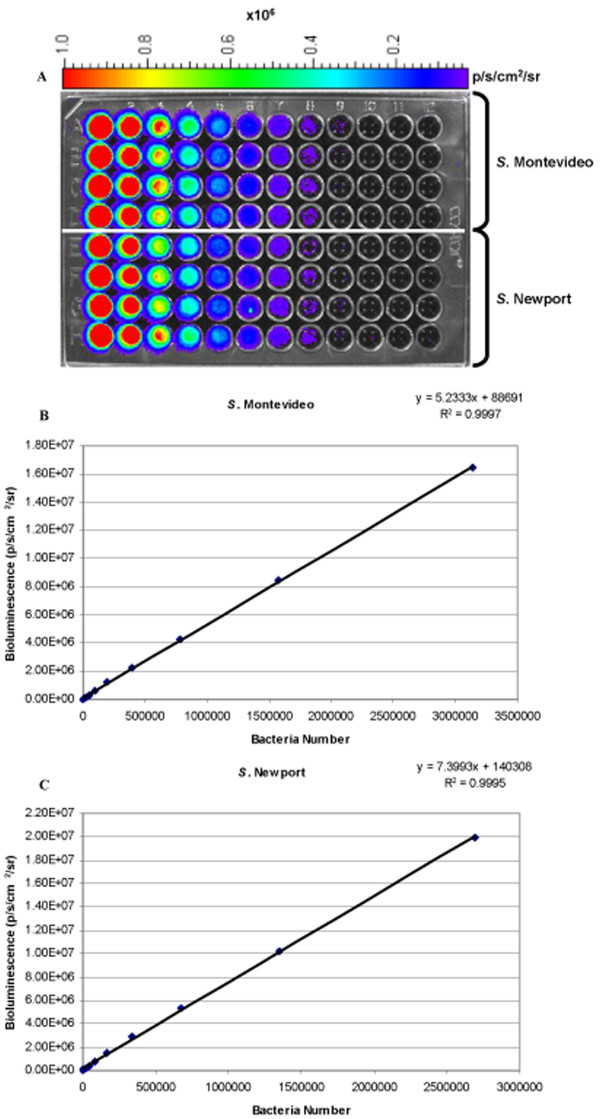
**Correlation between bioluminescence and bacterial numbers**. The correlation between luminescent signals and bacteria numbers for representative serovars *S*. Montevideo and *S*. Newport. A) 96-well plate containing *S*. Montevideo (rows A, B, C, and D) and *S*. Newport (rows E, F, G, and H). 25 μl of bacteria suspension from columns 11 and 12 were spread on LB agar plates with ampicillin (100 μg ml^-1^) to determine the concentration of colony forming units. B) plot of bioluminescence against bacterial numbers for *S*. Montevideo. C) plot of bioluminescence against bacterial numbers for *S*. Newport.

We showed that bacteria numbers and bioluminescence correlated well (*R*^2 ^= *0.99*) in all strains used (Figure 1). The minimum detectable numbers for all twelve strains was less than 1500 CFU/ml, and it was less than 300 CFU/ml for a majority of strains (*S*. Alachua, 334 CFU/ml; *S*. Braenderup, 217 CFU/ml; *S*. Enteritidis, 175 CFU/ml; *S*. Heidelberg, 169 CFU/ml; *S*. Kentucky, 229 CFU/ml; *S*. Mbandaka, 248 CFU/ml; *S*. Montevideo, 209 CFU/ml; *S*. Newport, 125 CFU/ml; *S*. Schwarzengrund, 1470 CFU/ml; *S*. Seftenberg, 1386 CFU/ml; *S*. Thompson, 1044 CFU/ml; and *S*. Typhimurium, 202 CFU/ml). This result was comparable to previous studies [13,15,29,30].

**Figure 2:**
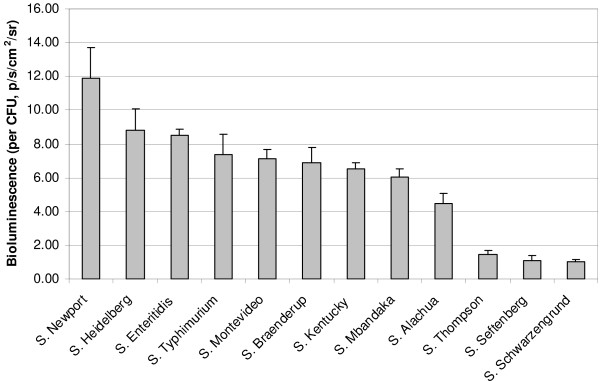
**Expression of bioluminescence in *Salmonella *serovars**. The theoretical amount of bioluminescence produced per CFU of each *Salmonella *serovar following 16 hrs of growth was calculated. Bioluminescence per cfu was calculated by dividing the background subtracted bioluminescence value (in p/s/cm^2^/sr) in each well by the number of bacteria (determined by serial dilution and plate counts). The mean and standard error from four replicates were then determined for each strain. Light emission was almost 12 fold higher in *S*. Newport as compared to *S*. Schwarzengrund.

The average theoretical light intensity per CFU was calculated for each strain and revealed a greater than 10-fold difference between some serovars (Figure 2). This difference was not due to lack of viability; bacterial plate counts showed that all the strains were viable at this stage and that viable bacterial densities for all the serovars were within a 3 fold range. The difference in luminescence could reflect a difference between serovars in efficiency of *luxCDABE *expression from the *lacZ *promoter, or it could reflect a difference between serovars in the activity of bacterial luciferase within the bacteria. Alternatively, the difference could reflect differences in metabolic activity between the serovars at this stage of growth (16 hours). Bioluminescence is known to correlate well with bacterial metabolism [31,32].

### Plasmid stability

**Figure 3:**
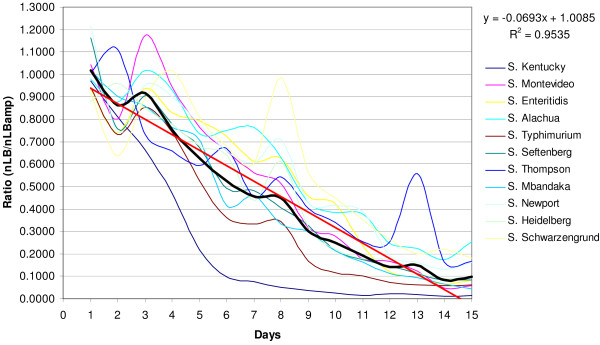
**Stability of pAK*lux*1 in *Salmonella***. *Salmonella *strains carrying pAK*lux*1 were subcultured under ampicillin selective and non-selective conditions for 15 days. At each passage, bioluminescence was measured and normalized for cell density (OD_600_). The ratio between the normalized values under non-selective (nLB) and ampicillin selected conditions (nLBamp) revealed the plasmid stability in different *Salmonella *serovars. The black line represents the mean of eleven strains, and the red line is the linear trend line.

The stability of pAK*lux*1 was determined by subculturing bioluminescent *Salmonella *broth cultures under selective (with ampicillin) and non-selective (no ampicillin) conditions for 15 days. Bioluminescence of *Salmonella *strains cultured under non-selective conditions was declining by day 2 and continued declining linearly (*R*^2 ^= *0.95*) until the conclusion of the experiment (Figure 3). Based on the data, the average half-life of pAK*lux*1 in *Salmonella *was approximately 7 days under the described culture conditions. However, the stability varied between *Salmonella *serovars. Among the *Salmonella *strains, plasmid stability was lower in *S*. Kentucky and *S*. Typhimurium as compared to the others. The half-life of pAK*lux*1 was about 4 days in *S*. Kentucky and about 5 days in *S*. Typhimurium (Figure 3). *S*. Newport, *S*. Slachua, and *S*. Enteritidis maintained pAK*lux*1 longer than others, with a half life of about 9 days.

pAK*lux*1 is derived from the broad host range plasmid pBBR1, which is relatively stable in gram-negative bacteria in the absence of antibiotic selection [33-35]. pAK*lux*1 was stable in the gram-negative species *Edwardsiella ictaluri *for at least 10 days without antibiotic selection, and the plasmid caused no alterations in growth kinetics, native plasmids, and pathogenicity as compared to the parent strain [15]. Data from the current study indicates that the pBBR1 replicon is not as stable in *Salmonella *as it is in other gram-negative bacteria. However, bioluminescent *Salmonella *strains labeled with pAK*lux*1 should be suitable for short term experiments in which antibiotic selection cannot be applied.

### Characterization of skin attachment properties of *Salmonella *strains

**Figure 4:**
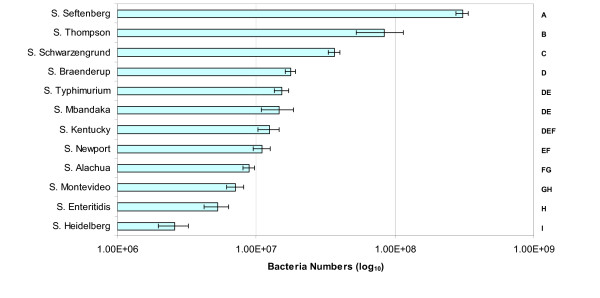
**Attachment of *Salmonella *strains to chicken skin**. The amount of *Salmonella *attachment to chicken skin was determined by measuring the bioluminescence following 1 h incubation of bacterial suspension with quadruplicate skin samples. Bacteria numbers of each strain were estimated from the bioluminescence values using each strain's linear correlation formula. Unattached *Salmonella *were removed by gentle washing prior to bioluminescence measurements. The bacteria number for each strain is the geometric mean of four replicates from three separate experiments. Letters on the right of the graph indicate statistical groupings as determined by Tukey's test. Values with the same letter are not significantly different (*P *> 0.05).

We developed an *in vitro *skin attachment model for characterization of attachment properties of different *Salmonella *strains using BLI. Bioluminescence was successfully detected on chicken skin after being exposed to *Salmonella *strains expressing pAK*lux*1. Using this model, we were able to show that *Salmonella *strains from different serovars vary in their ability to attach to chicken skin (Figure 4). Bacteria numbers in *S*. Seftenberg, *S*. Thompson, and *S*. Schwarzengrund were significantly different from each other and from other strains (*P *≤ 0.05). The *S*. Heidelberg strain had significantly less binding to chicken skin than all the other strains and was 79 fold lower than *S*. Seftenberg, which had the highest amount of binding (Figure 4). This result suggests that *Salmonella *strains vary in their ability to bind to chicken skin; this ability may be a discriminating factor determining whether *Salmonella *strains persist through processing or whether they are removed.

### The effect of washing on *Salmonella *removal from chicken skin

**Figure 5:**
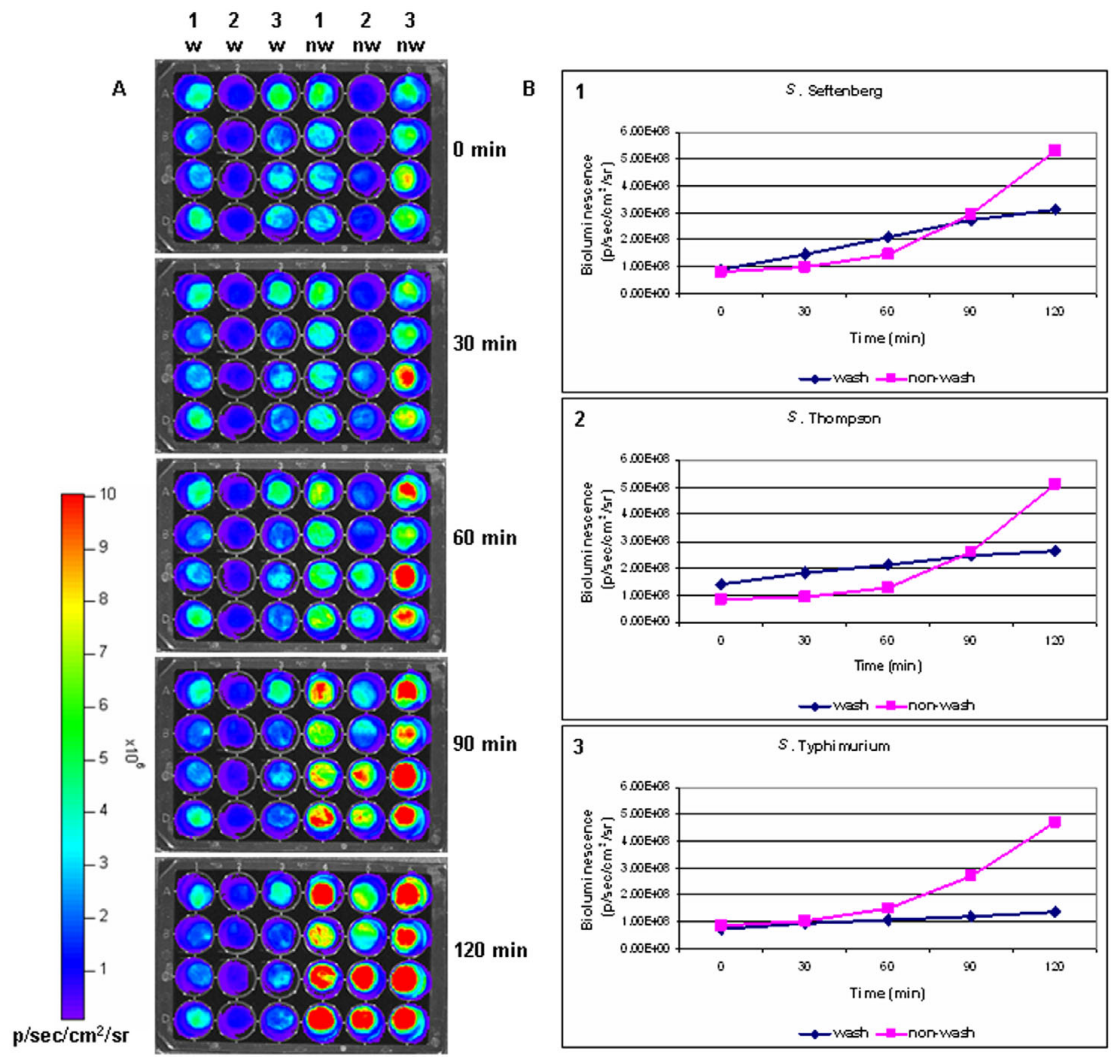
**The effect of washing on removal of *Salmonella *from chicken skin**. Bioluminescence was used to measure the ability of agitated water bath to remove *Salmonella *strains from chicken skin. *Salmonella *was allowed to attach to chicken skin for 1 h, and then the amount of *Salmonella *present on skin that received four 30 min washes was compared to the amount of *Salmonella *present on non-washed skin. Bioluminescence was measured after each 30 min wash, and the mean of four replicates from three separate experiments was determined. A) Representative plate containing 3 *Salmonella *strains at five time points. Half of the plate included washed skin samples (w) and the other half included unwashed skin controls (nw). Column 1 is *S*. Seftenberg, column 2 is *S*. Thompson, and column 3 is *S*. Typhimurium. B) Amount of bioluminescence for the three representative strains at each time point.

Bioluminescence was an effective tool for measuring the effects of washing for removal of *Salmonella *from chicken skin using our model (Figure 5). Bioluminescent *Salmonella *strains have been previously utilized to monitor progress of infection, effect of heat and pH treatments, growth in food samples, and toxicity [13,20,22,36]. One study used a single bioluminescent *Salmonella *Hadar isolate to investigate the effectiveness of washing for removal of *Salmonella *from turkey skin [20]. The current study demonstrates, for the first time, use of BLI for real-time monitoring of twelve *Salmonella *strains using a chicken skin model.

**Figure 6:**
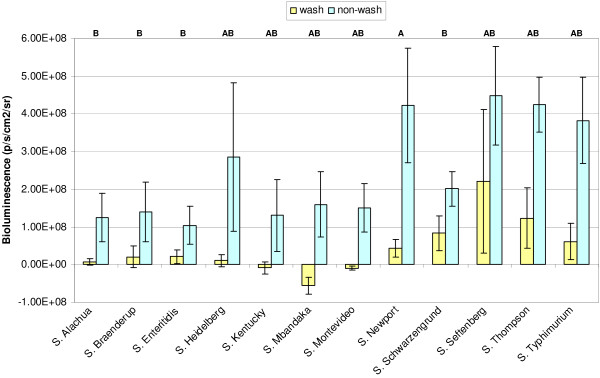
**Comparison of the amount of *Salmonella *before and after 2 h incubation**. The change in bioluminescence on chicken skin following 2 h incubation (relative to pre-wash bioluminescence) for washed and non-washed treatments is shown. Letters at the top of the graph indicate statistical groupings for the mean differences between washed and non-washed treatments within each strain as determined by Tukey's test. Values with the same letter are not significantly different (*P *> 0.05).

As a general trend observed from all strains, washing suppressed the reproduction of *Salmonella *on chicken skin, probably due to physical removal of bacteria (Figure 6). In non-washed skin samples, *Salmonella *numbers increased steadily over the two hour incubation period (Figure 5B), with final numbers showing an increase ranging from 143% (*S*. Schwarzengrund) to 459% (*S*. Newport) compared to initial measurement at time zero. In washed skin samples, the increase in *Salmonella *numbers was lower, demonstrating the effectiveness of simple agitation in water for suppressing *Salmonella *growth on chicken skin.

For all 12 strains, the washed treatments had a significantly lower change in bioluminescence than the corresponding non-washed treatments after the 2 h wash period. However, there was strain variation in the effectiveness of washing (Figure 6). For three strains (*S*. Kentucky, *S*. Mbandaka and *S*. Montevideo), washing reduced the number of *Salmonella *present on chicken skin at 120 min compared to 0 min (pre-wash). For the other strains, although washing did not decrease the number of *Salmonella *on chicken skin, it was effective in significantly reducing the growth relative to non-washed samples.

When the differences in bioluminescence between washed and non-washed treatments at 120 min were compared across strains, the decrease in bioluminescence for *S*. Newport caused by washing was significantly greater than the decrease in bioluminescence for *S*. Alachua, *S*. Braenderup, *S*. Enteritidis, and *S*. Schwarzengrund (Figure 6). Thus, based on the difference in bioluminescence between washed and non-washed treatments, washing was apparently most effective in removing *S*. Newport and least effective for *S*. Alachua, *S*. Braenderup, *S*. Enteritidis, and *S*. Schwarzengrund. However, it is interesting to note that the two strains that attached to chicken skin most effectively (*S*. Seftenberg and *S*. Thompson; Figure 4) were also able to increase their numbers in the wash treatment most effectively (3.7 and 2.7 fold increases, respectively, relative to pre-wash) over the 2 hr incubation.

## Conclusion

Our results demonstrate that pAK*lux*1 is effective for labeling *Salmonella enterica *strains with bioluminescence. Using this tool, we were able to develop twelve bioluminescent *Salmonella *strains that were isolated from the poultry production/processing continuum. However, stability results indicated that pAK*lux*1 is not as stable in *Salmonella *as it is in other gram-negative species. Therefore, this labeling system may not be useful for long-term experiments, and work is already in progress in our group to develop a labeling system for *Salmonella *that is more stable.

Our work also shows that bioluminescence is an effective and sensitive method to monitor *Salmonella *on food products. In particular, our study demonstrates that *Salmonella *strains vary in their ability to attach to chicken skin. Future research aimed at determining whether the ability to attach to chicken skin correlates with the ability to persist in poultry production/processing environments is warranted. Finally, our results indicate that simple washing in an agitated water bath can remove contaminating *Salmonella *from chicken skin, but this method alone cannot eliminate the *Salmonella *completely due to strong bacterial attachment to skin. Therefore, additional methodologies should be used in poultry processing to eliminate *Salmonella *from the chicken skin to prevent disease outbreaks. The skin model developed in this study should be very useful for testing alternative strategies in addition to washing procedures.

## Methods

### Bacterial strains and growth media

As part of a previous study, we have collected, isolated, and serotyped thousands of *Salmonella *specimens derived and cataloged at five different sites along the broiler production continuum: post hatchery, prior to harvest, arrival at the plant, pre-chill tank, and post-chill tank. *Salmonella *isolates were collected from 66 different flocks during the years 2003–2006. The cataloged information ascribed to each isolate includes location within the production continuum, flock environmental and production parameters, as well as processing plant information. Strains from twelve *Salmonella enterica *serovars from the poultry production continuum were selected for this study (*S*. Alachua, *S*. Braenderup, *S*. Enteritidis, *S*. Heidelberg, *S*. Kentucky, *S*. Mbandaka, *S*. Montevideo, *S*. Newport, *S*. Schwarzengrund, *S*. Seftenberg, *S*. Thompson, and *S*. Typhimurium). *Salmonella *strains were grown using Luria-Bertani broth and agar plates at 37°C, and bioluminescent *Salmonella *strains were grown in the same medium containing ampicillin (100 μg ml^-1^) for plasmid maintenance.

### Bioluminescence tagging of *Salmonella *strains

Bioluminescent *Salmonella enterica *strains were established using the broad host range plasmid pAK*lux*1 containing the *luxCDABE *operon from *Photorhabdus luminescens *[15]. *Salmonella *strains were grown to logarithmic phase (OD_600 _of 0.6–0.8), made electrocompetent by washing with 10% cold glycerol solution four times, and stored at -80°C. pAK*lux*1 was isolated from *Escherichia coli *DH5α strain by QIAprep Spin Miniprep Kit (Qiagen, Valencia, CA). *Salmonella *strains were transformed with pAK*lux*1 by electroporation using a Gene Pulser II system at 2.5 kV, 25 uF, and 400 Ω (Bio-Rad, Hercules, CA). Cells were allowed to recover for 1 h at 37°C in SOC media (Invitrogen Corp., Carlsbad, CA). Following recovery, bacteria were spread on LB plates with ampicillin and placed in an incubator at 37°C for approximately 16 h. Ampicillin resistant bioluminescent *Salmonella *colonies were detected using a ChemiImager 5500 imaging system with AlphaEaseFC software (Alpha Innotech, San Leandro, CA) or using an IVIS Imaging System 100 Series with Living Image Software v2.50 (Xenogen Corp., Alameda, CA).

### Characterizing the bioluminescence properties of *Salmonella *strains

Bioluminescent *Salmonella *strains were grown overnight, and OD_600 _values of each strain were measured from quadruplicate samples in a 96-well plate using ThermoMax spectrometer (Molecular Devices, Sunnyvale, USA). Following OD_600 _measurements, four separate dilution series were prepared from each strain in black 96-well microtiter plates. Each series contained 2 × 10^-3^, 4 × 10^-3^, 8 × 10^-3^, 1.6 × 10^-4^, 3.2 × 10^-4^, 6.4 × 10^-4^, 1.3 × 10^-5^, and 2.6 × 10^-5 ^dilutions. Bioluminescence was measured for 5 s at 37°C using an IVIS Imaging System 100 Series, and bioluminescence was quantified using Living Image software v2.50 (Xenogen Corp.). The last two dilutions were spread on LB agar with ampicillin to determine viable bacterial densities.

The linear correlation between population densities and bioluminescence was determined for each strain by plotting bioluminescence against bacteria numbers determined by duplicate plate counts. The minimum detectible number for each strain was determined using the number of bacteria present in the last dilution that had detectable luminescence above background. Colony counts were also used to calculate the theoretical amount of bioluminescence produced per CFU for each strain, which was used to compare expression of bioluminescence among serovars.

### Plasmid stability *in vitro*

Plasmid stability of pAK*lux*1 in eleven *Salmonella *strains was analyzed by subculturing bioluminescent *Salmonella *strains in LB medium with and without ampicillin 15 times. For each passage, new 0.1 ml cultures were inoculated in microplates in quadruplicate from 16 h cultures at a 40-fold dilution. Bacterial density (OD_600_) and bioluminescence were determined for each of the 16 h cultures prior to starting new subcultures. Bioluminescence was measured using an IVIS Imaging System for 5 s at 37°C and normalized by dividing total flux by OD_600 _readings. The average normalized bioluminescence for each strain and passage was determined under non-selective and ampicillin-selected conditions, and plasmid stability was determined by calculating the ratio between normalized bioluminescence under non-selective conditions versus ampicillin-selected conditions. This revealed the capability of each serovar to maintain pAK*lux*1 under non-selective conditions.

### Development of skin attachment model

An experimental model for investigating bacterial attachment to chicken skin was established. Chicken skin was obtained from a commercial poultry processing plant and submerged in 0.26% sodium hypochlorite solution for approximately 2 h and stored at 4°C until use. Circular sections of skin approximately 8 mm in diameter were made using a circular cutting blade and placed in black 24-well culture plates. To determine the optimal bacterial dose to prevent loss of experimental data due to image saturation, chicken skin was incubated with different numbers of bioluminescent *Salmonella *(1 × 10^8 ^to 2.5 × 10^4 ^CFU) in 1 ml of phosphate-buffered saline. Based on these results, we used a bacterial concentration of 1 × 10^6 ^CFU/ml for subsequent experiments because this dose does not cause saturation and because it falls in the linear detection interval (1 × 10^3^-1 × 10^8 ^photons/s/cm^2^/steradean [p/s/cm^2^/sr]).

### Characterization of skin attachment properties of *Salmonella* strains

After establishing the skin attachment model, differential attachment properties of twelve *Salmonella *strains were determined. For each strain, overnight bioluminescent *Salmonella *cultures were diluted to approximately 1 × 10^6 ^CFU/ml in distilled water, and 1 ml was added to skin sections in quadruplicate. Plates were incubated at room temperature for 1 h to allow *Salmonella *to attach to skin. Then bacterial suspension was removed, and each well was gently washed twice with distilled water to remove unattached bacteria. Immediately after washing, plates were warmed to 37°C for 5 min and bioluminescence was measured for 15 s using the IVIS Imaging System. The experiment was repeated three times.

Total flux was calculated from the pseudo color images. To normalize for bacterial density differences between strains, the total flux from each well was divided by the OD_600 _of the bacterial suspension used to infect that well. Because the amount of bioluminescence from different strains varied, theoretical bacterial numbers were calculated from bioluminescence using the linear correlation formulas calculated for each strain (Figure 1). The data were transformed by taking the base 10 logarithm of the calculated bacterial numbers to improve normality. To compare bacterial numbers of the 12 *Salmonella *strains following skin attachment (after 1 h incubation), a two-way analysis of variance (ANOVA) of the transformed data was conducted using PROC GLM SAS 9.1 (SAS Institute Inc., Carey NC). Variables for replicate, strain, and their interaction were included in the model. Pairwise comparison of the means was done using Tukey procedure. A significance level of *P *≤ 0.05 was used. Data were then retransformed to percent mortality for interpretation.

### The effect of washing on *Salmonella* removal from chicken skin

The effect of washing chicken skin in an agitated water bath for removal of *Salmonella *was determined. The same 12 bioluminescent *Salmonella *strains were allowed to attach to chicken skin sections using the exact method described for the attachment experiment. In this experiment, two treatments were included for each strain: a washed treatment and a control (non-washed) treatment. Each treatment was set up in quadruplicate, and the experiment was repeated three times. Each bioluminescence measurement was conducted after warming plates to 37°C for 5 min. Following the 1 h incubation for attachment and washes to remove unattached bacteria, bioluminescence was measured. One ml of distilled water was added to the washed treatments, and control treatments received no water. Plates were incubated at room temperature for 30 min with agitation at 200 rpm. Water was removed from the washed treatments, and bioluminescence was measured. One ml of water was added to the washed skin treatments, and the wash procedure was repeated three more times (2 h total). After each wash, bioluminescence was measured. Total flux was measured from the pseudo color images for each time point, and samples were normalized as described for the attachment experiment.

To determine the effect of washing within each strain, the mean difference in bioluminescence between 0 minutes (prior to washing) and 120 minutes was calculated for both washed and non-washed treatments for each strain. The mean differences of washed and non-washed treatments within each strain were then compared by conducting a two-way analysis of variance using PROC GLM SAS 9.1 (SAS Institute Inc., Carey NC) for each strain. Variables for replicate, treatment, and their interaction were included in each model. To compare the effect of washing across strains, the mean difference in bioluminescence between washed and non-washed treatments after 120 minutes was calculated for each strain. A two-way analysis of variance was conducted on the mean differences using PROC GLM SAS 9.1 (SAS Institute Inc., Carey NC). Variables for replicate, strain, and their interaction were included in the model. Pairwise comparison of the means was done using Tukey procedure. A significance level of 0.05 was used in all analyses.

## Authors' contributions

HB and RW isolated the *Salmonella *strains. ML, HB, and AK designed the bacteriological and genetic studies. AK, KH, and TK performed the experiments and analyses. RW and AK designed and conducted statistical analyses. AK, KH, ML, and HB drafted the manuscript. All authors read and approved the final manuscript.
